# Salivary Interleukin-6 and Interleukin-18 Levels and Their Association with Dental Health in Children with Idiopathic Nephrotic Syndrome

**DOI:** 10.3390/ijms26073175

**Published:** 2025-03-29

**Authors:** Paula Piekoszewska-Ziętek, Natalia Korytowska-Przybylska, Małgorzata Pańczyk-Tomaszewska, Dorota Olczak-Kowalczyk

**Affiliations:** 1 Department of Pediatric Dentistry, Medical University of Warsaw, 02-091 Warsaw, Poland; 2 Department of Drug Chemistry, Pharmaceutical and Biomedical Analysis, Medical University of Warsaw, 02-091 Warsaw, Poland; natalia.korytowska-przybylska@wum.edu.pl; 3 Department of Pediatrics and Nephrology, Medical University of Warsaw, 02-091 Warsaw, Poland; mpanczyk1@wum.edu.pl

**Keywords:** nephrotic syndrome, oral diseases, caries, interleukin 6, interleukin 18

## Abstract

Idiopathic nephrotic syndrome (NS) is associated with immune dysfunction and increased susceptibility to infections. Oral health may influence systemic inflammation and disease progression. This study aimed to evaluate the salivary levels of interleukin-6 (IL-6) and interleukin-18 (IL-18) in children with NS and their association with dental health, particularly caries prevalence and the consequences of untreated caries. A cross-sectional study was conducted on 86 children aged 5–17 years, including 40 NS patients and 46 healthy controls. Clinical dental examinations assessed caries prevalence using the dmft/DMFT index and the impact of untreated caries using the pufa/PUFA index. Unstimulated saliva samples were collected, and IL-6 and IL-18 concentrations were measured using enzyme-linked immunosorbent assay. NS patients exhibited a significantly lower prevalence of active carious lesions than controls (50% vs. 72%, *p* = 0.039). The DMFT index was lower in the NS group (*p* = 0.003). Salivary IL-6 levels were significantly reduced in NS patients compared to controls (*p* = 0.015), while IL-18 levels showed no significant difference. IL-6 positively correlated with decayed permanent teeth and pulp/periapical tissue diseases, whereas IL-18 correlated with white spot lesions and pulp infections. IL-6 and IL-18 could serve as potential non-invasive indicators of disease progression in NS patients.

## 1. Introduction

Idiopathic nephrotic syndrome is the most frequently occurring glomerular disease in children [1]. The annual incidence of nephrotic syndrome (NS) is estimated to range between 1 and 4 cases per 100,000 children, varying depending on age, ethnicity, and geographical location. While the condition can manifest as early as the first year of life, it most commonly develops between 2 and 7 years of age [2]. NS has a tendency to recur, with relapses being triggered by various factors. These may include infections (especially respiratory and urinary tract infections), vaccinations, allergic reactions, stress, and non-compliance with treatment [3,4]. Additionally, oral health conditions, such as untreated dental caries, periodontal disease, and other inflammatory processes in the oral cavity, can contribute to disease exacerbation by acting as a source of chronic infection and systemic inflammation [5].

Impairment of the body’s defense mechanisms in nephrotic syndrome (primarily cellular immune response) is mainly a consequence of hyperlipidemia and the effects of immunosuppressive therapy, including glucocorticoids, cyclosporine A, cyclophosphamide, and, less frequently, mycophenolate mofetil [6]. Reduced immunity predisposes patients to bacterial, viral, and fungal infections. Furthermore, infections are a significant risk factor for disease relapse. Therefore, in children with nephrotic syndrome, preventing infections is crucial, and when they occur, they must be treated intensively. In the event of a disease relapse, it is essential to exclude potential sources of infection, including those that may be present in the oral cavity [7].

Studies have demonstrated that oral infections can influence the pathogenesis of systemic conditions. For instance, research indicates that periodontal disease is associated with elevated systemic inflammatory markers, including IL-6 and TNF-α. Additionally, the secretion of PGE2, IL-1β, IL-6, and TNF-α by mononuclear cells in response to lipopolysaccharides from periodontal pathogens underscores the systemic inflammatory potential of oral infections. Therefore, maintaining oral health is essential not only for preventing local diseases but also for mitigating the risk of systemic complications [8,9]. Interleukin-18 (IL-18), a member of the IL-1 cytokine superfamily, is a potent pro-inflammatory cytokine that plays a crucial role in regulating both innate and adaptive immune responses. Produced by various cell types, including monocytes, macrophages, keratinocytes, and mesenchymal cells, IL-18 is instrumental in host defense against infections [10]. Given its central role in immune modulation, IL-18 has become a target for therapeutic interventions aimed at modulating its activity in various disease states [10].

Patients with nephrotic syndrome (NS) commonly exhibit a range of oral manifestations, including enamel hypoplasia, enamel opacities, increased calculus deposition, anatomical malformations of teeth or soft tissue calcification [11]. While the diagnosis and management of mucosal lesions, dental caries, and periodontal conditions are fundamental aspects of dental care, there is a notable lack of research on the oral health status of children with nephrotic syndrome. Given the dispersed nature of existing reports and the limited data available, the present study aims to assess the salivary concentration of interleukin 6 and interleukin 18 in terms of the prevalence of dental caries and clinical consequences of untreated caries in children with idiopathic nephrotic syndrome.

## 2. Results

The study included 86 patients, with 40 in the study group (nephrotic syndrome) and 46 in the control group (generally healthy individuals). The study group comprised 28 boys (70%) and 12 girls (30%), while the control group included 24 boys (52%) and 22 girls (48%). The mean age of the patients was 11.84 ± 4.09 years. No significant differences were observed between the study and control groups regarding age and sex distribution (*p* > 0.05). Detailed data on the type of dentition in the study participants are presented in Table 1.

In the study group, 34 cases of caries were recorded (85%), compared to 43 cases in the control group (93%) (*p* = 0.293). However, a significant difference was observed in the number of patients with active carious lesions, with 20 cases (50%) in the study group and 33 cases (72%) in the control group (*p* = 0.039).

Regarding primary teeth, the study group showed similar or lower values of the dmft index and its components (d—decayed milk teeth; m—missing milk teeth). The number of filled primary teeth was higher in the study group. Minor differences were also noted in the mean number of teeth with white spot lesions. However, none of these differences related to primary teeth were statistically significant. A summary of the data is presented in Table 2.

For permanent teeth, the study group had lower values of the DMFT index and its individual components (D—decayed permanent teeth; M—missing permanent teeth; F—filled permanent teeth). Statistically significant differences were found in the mean number of permanent teeth with active carious lesions (0.97 ± 1.56 vs. 3.77 ± 5.18; *p* = 0.003) and the total DMFT index value (3.43 ± 4.12 vs. 7.02 ± 6.3; *p* = 0.003). Additionally, the study group had a lower mean number of permanent teeth with white spot lesions. Detailed data are provided in Table 2.

In the study group, 8 (20%) cases of pulp and periapical tissue diseases were recorded, compared to 17 (37%) cases in the control group (*p* = 0.084). The pufa index for primary dentition did not show significant differences between the study and control groups. However, for permanent teeth, a significant difference was observed in the presence of severely decayed teeth with visible pulpal involvement (P component) (0.03 ± 0.17 vs. 0.53 ± 1.24; *p* = 0.009). The details are described in Table 3.

Patients with nephrotic syndrome exhibited a significantly lower level of salivary interleukin 6 (IL-6) and no differences in salivary interleukin 18 (IL-18) concentration (Table 4).

Spearman’s rank correlation analysis revealed a significant positive correlation between salivary IL-6 concentration and the presence of carious permanent teeth (D component of the DMFT index), as well as pulp diseases and their complications in the form of periapical tissue inflammation. Additionally, a significant positive correlation was observed between salivary IL-18 concentration and the occurrence of white spot lesions (ICDAS-II codes 1–2) as well as pulp and periapical tissue diseases. Detailed data are presented in Table 5.

## 3. Discussion

The study provides constructive insights into the oral health status of children with idiopathic nephrotic syndrome (NS) and its potential relationship with systemic inflammation. While caries prevalence was high in both groups (85% in the study group vs. 93% in the control group), the number of patients with active carious lesions was significantly lower in children with NS (50% vs. 72%). The results are in contrast with previous studies that evaluated the incidence of dental caries in patients with NS, particularly among children. These studies have consistently reported a higher prevalence of dental caries in this population compared to healthy controls [7,12. Vietnamese research involving over 800 children with primary nephrotic syndrome and controls found that the NS group had a significantly higher prevalence of dental caries. Specifically, the caries rate in the NS group was 90.7%, with a mean decayed, missing, and filled teeth (dmft) index of 3.98, compared to lower rates in the control group [12]. However, there is other research that reports the outcome of reduced caries prevalence among patients with nephrotic syndrome or other renal diseases [13,14]. In the study by Babu et al. [13], 11% of children with permanent dentition and 17% of children with primary dentition had dental caries, which is lower than the result achieved in the healthy population. Another Polish study [14] found a lower caries experience in the NS group (83.0%) compared to the controls (95.7%). A recent study published in 2025 by Beyer et al. [15] examined the oral health of children with chronic kidney disease, nephrotic syndrome, and those who received kidney transplants. The study found that children with renal conditions had poorer oral health compared to healthy controls, highlighting the need for comprehensive dental care in these populations.

Babu et al. [13] suggest that the lower caries prevalence in children with nephrotic syndrome may be attributed to elevated salivary pH due to increased urea levels, which neutralize bacterial acid production, inhibit plaque formation, and enhance remineralization due to high phosphate concentrations. While this biological mechanism may play a role, our study suggests an additional contributing factor: proactive dental care. At our institution, close cooperation with nephrologists ensures that children with nephrotic syndrome receive regular dental check-ups, preventive education, and necessary treatments. This structured approach not only reduces the incidence of active carious lesions but also helps mitigate the risk of disease relapse associated with untreated caries. Unlike previous studies that report higher caries prevalence in NS patients, our findings highlight the impact of interdisciplinary care in improving oral health outcomes in this vulnerable population.

The discrepancies in reported caries prevalence among children with nephrotic syndrome across various studies may stem from several factors. Differences in patient populations—such as age, disease duration, and comorbidities—can influence oral health outcomes [12,15]. Variability in immunosuppressive regimens, including drug type, dosage, and cumulative exposure, may also affect salivary flow and immune function, thereby modifying caries risk [13]. For example, Babu et al. [13] hypothesized that reduced caries risk may be due to elevated salivary urea and pH in NS patients, while Luong et al. [12] reported increased caries in a population with limited access to dental services. Socio-economic and cultural factors that impact diet and oral hygiene practices should also be considered [14]. Finally, methodological differences in caries detection criteria, clinical calibration, and sample size may affect comparability between studies. Taken together, these elements highlight the complexity of interpreting caries risk in this patient population and the necessity for harmonized research protocols [15].

To the best of our knowledge, there are no available studies that assess indices related to the consequences of untreated caries in patients with nephrotic syndrome, such as the pufa/PUFA index. Existing research has primarily focused on evaluating the overall prevalence of dental caries and general oral health status in this patient group. However, there is a lack of detailed analyses regarding the advanced stages of caries and their potential impact on the course of nephrotic syndrome. Our study provides a significant contribution to this topic, emphasizing the necessity of an interdisciplinary approach to dental care for nephrology patients.

In the presented study, NS patients exhibited significantly lower levels of salivary interleukin-6 (IL-6), whereas IL-18 levels did not differ significantly between the groups. Lower IL-6 levels could suggest immune dysregulation or suppression related to nephrotic syndrome or its treatment, potentially affecting oral disease progression and systemic inflammatory responses. IL-6 is a cytokine involved in inflammatory responses and has been implicated in various oral diseases. A review discussed the role of IL-6 in oral diseases, noting that elevated IL-6 levels are associated with conditions such as periodontal disease and oral cancer. Conversely, lower IL-6 levels could suggest a diminished inflammatory response, potentially affecting the progression of oral diseases [16].

Research has been conducted to analyze salivary interleukins in patients with nephrotic syndrome. A notable study compared salivary cytokines and total protein levels between children with NS and healthy controls. The findings revealed that children with NS had significantly lower salivary levels of total protein, interleukin-6 (IL-6), and interleukin-8 (IL-8) compared to healthy children, which confirms the findings of our study. Additionally, salivary protein and IL-8 levels were lower during relapse than during remission. These results suggest that salivary parameters, including cytokine levels, may reflect systemic changes in NS and could potentially serve as non-invasive biomarkers for monitoring disease activity [17].

In our research, IL-6 levels positively correlated with the presence of carious permanent teeth and pulp/periapical diseases, reinforcing its role as an inflammatory marker in oral infections [18]. IL-18 levels showed a significant positive correlation with white spot lesions and pulp/periapical diseases. The observed significant correlation suggests a possible link between subclinical inflammation and early caries development. However, this remains a hypothesis that requires further confirmation. While previous studies have associated IL-18 with periodontal inflammation and systemic conditions, there is currently no direct evidence linking IL-18 to the initial stages of caries progression [19]. Future research is necessary to clarify the role of IL-18 in early caries development and its potential as a biomarker for oral inflammatory processes.

The results of our study suggest that salivary IL-6 and IL-18 may serve as non-invasive adjunctive biomarkers to assess inflammatory activity in children with NS, particularly in the context of oral health status. While these cytokines are not currently standard components of NS monitoring, their use could be piloted in clinical settings with high-risk patients—such as those with frequent relapses or poor oral hygiene—by integrating salivary testing into routine dental check-ups or nephrology follow-up appointments. Point-of-care saliva tests are under development in other medical fields and may eventually provide real-time results to guide clinical decisions. In our center, a collaborative care model has been implemented whereby children with nephrotic syndrome are referred for dental screening at the time of diagnosis and monitored every 3–6 months thereafter. This model involves close communication between nephrologists and pediatric dentists, including shared medical histories and access to laboratory findings. Such a system enables timely dental intervention, which may reduce the risk of infection-related relapses and improve overall health outcomes. Expanding this model to include salivary biomarker screening could further enhance the early detection of oral or systemic inflammation.

This study has several limitations that should be acknowledged. First, its cross-sectional design prevents establishing causal relationships between salivary cytokine levels and oral health parameters in children with nephrotic syndrome. The relatively small sample size may limit the generalizability of the findings, and larger multicenter studies would be needed to confirm these results. Although none of the participants were undergoing immunosuppressive therapy at the time of the study, we recognize that previous exposure—especially repeated or prolonged use—may still affect immune responses and salivary cytokine levels. This potential confounding factor should be taken into account in future studies, ideally through stratification based on treatment duration, type, and proximity to saliva collection. The study also did not account for dietary habits, oral hygiene practices, fluoride exposure, or socio-economic status, all of which could influence caries prevalence and may potentially modulate salivary biomarker levels. Future studies should incorporate these factors to provide a more comprehensive understanding of the determinants of oral health in children with NS. As a cross-sectional study, our findings can only demonstrate associations rather than causality between cytokine levels and dental outcomes. Longitudinal studies following patients over time, particularly across disease relapses and remissions, are needed to clarify the temporal relationship between nephrotic syndrome activity, immune dysregulation, and oral inflammatory status.

Oral health plays a critical role in systemic inflammation, especially in immunocompromised children with nephrotic syndrome. Given the correlation between salivary cytokine levels and caries activity, salivary biomarkers such as IL-6 and IL-18 could serve as potential non-invasive indicators of disease progression in NS patients. Beyond identifying salivary IL-6 and IL-18 as potential biomarkers, our findings highlight the value of establishing structured, interdisciplinary care pathways involving both nephrologists and dental professionals. Implementing routine dental assessments and exploring the integration of salivary biomarker screening may help reduce relapse triggers and improve the quality of care for pediatric NS patients. Further studies should explore the long-term effects of nephrotic syndrome on dental health, the impact of immunosuppressive therapy on oral microbiota, and potential preventive strategies tailored for NS patients.

## 4. Materials and Methods

A favorable opinion for this study was given by the Medical University of Warsaw’s Bioethical Commission (KB/26/2020). The study is in compliance with STROBE guidelines and the Declaration of Helsinki principles.

### 4.1. Study Design and Participants

This cross-sectional study included patients aged from 5 to 17 years of age, divided into two groups: the study group consisting of patients diagnosed with nephrotic syndrome and the control group comprising generally healthy individuals. The inclusion criteria for the study group were a confirmed diagnosis of nephrotic syndrome, no ongoing immunosuppressive treatment at the time of examination, and the absence of other systemic diseases. The control group included healthy individuals with no history of nephrotic syndrome or other chronic systemic diseases. Exclusion criteria for both groups encompassed antibiotic use within the previous three months, systemic conditions affecting salivary secretion and any chronic medication intake. Written consent from the child and/or his parents/legal guardians was collected prior to the study. Patients who did not fully comply with all the criteria were excluded from participation.

### 4.2. Clinical Examination

All participants underwent a comprehensive intraoral examination conducted by a single calibrated examiner (intraexaminer reliability κ = 0.92) under standardized conditions. The clinical evaluation included assessments of caries prevalence using the dmft/DMFT index (decayed, missing, and filled teeth for primary and permanent dentition) [20], the presence of active carious lesions, and the number of white spot lesions (ICDAS-II codes 1–2) [21]. Pulpal and periapical tissue diseases were diagnosed based on clinical symptoms, and the pufa/PUFA index (pulpal involvement, ulceration, fistula, and abscess) [22] was recorded for both primary and permanent teeth.

### 4.3. Saliva Collection and Biomarker Analysis

All saliva samples were collected in the morning between 8:00 and 10:00 a.m. to control for circadian variability. Participants refrained from food and beverage intake for at least one hour before collection. Unstimulated whole saliva was obtained using Salivette collection tubes (Sarstedt AG & Co., Nümbrecht, Germany) over a 5-min period under supervision. Samples were centrifuged immediately after collection following the manufacturer’s protocol, and the supernatant was frozen within 30 min at −80 °C. All samples were analyzed in a single freeze cycle to avoid the degradation of biomarkers due to multiple freeze-thaw events. Salivary concentrations of interleukin 6 (IL-6) and interleukin 18 (IL-18) were measured using enzyme-linked immunosorbent assay (ELISA) kits obtained from Thermo Fisher Scientific (Human IL-18 ELISA Kit cat. no. BMS267-2, Human IL-6 High Sensitivity ELISA Kit, cat. no. BMS213-2HS) (Waltham, MA, USA) according to the manufacturer’s instructions.

### 4.4. Statistical Analysis

For quantitative data, Student’s t-tests for independent samples were used when the data did not exhibit significant skewness; otherwise, the Mann-Whitney U test was applied. For results reaching statistical significance or showing a trend toward significance, the effect size was calculated using Cohen’s d or the r coefficient, respectively. For categorical or ordinal data, chi-square tests (χ^2^) were performed, while Fisher’s exact test was used when the assumptions of the chi-square test were not met. For statistically significant results or those at the trend level, the effect size was determined using Cramer’s V coefficient. Additionally, Spearman’s rank correlation analysis was conducted to assess associations between selected variables. A significance level of *p* < 0.05 was considered statistically significant.

## Figures and Tables

**Table 1:** Dentition type distribution in patients included in the study.

Dentition Type	Control Group (n = 46) n/%	Study Group (n = 40) n/%	Statistical Significance
Primary	3/7	5/12	*p* = 0.464
Mixed	11/24	17/43	*p* = 0.067
Permanent	32/69	18/45	*p* = 0.021

**Table 2:** Differences in dmft/DMFT index and white spot lesion score (ICDAS-II 1–2) in the study and control groups.

	Control Group (n = 46)	Study Group (n = 40)	Statistical Significance
Mean number of primary teeth with ICDAS-II 1–2 score	1.86 ± 1.88	1.41 ± 1.40	Z = −0.54; *p* = 0.593
Mean d	3.14 ± 3.41	1.45 ± 2.20	Z = −1.86; *p* = 0.062; r = 0.31
Mean m	0.71 ± 1.9	0.73 ± 1.72	Z = −0.11; *p* = 0.911
Mean f	1.43 ± 1.56	1.64 ± 2.15	Z = 0; *p* = 1
Mean dmft	5.29 ± 3.65	3.81 ± 3.66	Z = −1.24; *p* = 0.214
Mean number of permanent teeth with ICDAS-II 1–2 score	1.74 ± 2.41	1.23 ± 1.63	Z = −0.88; *p* = 0.376
Mean D	3.77 ± 5.18	0.97 ± 1.56	Z = −2.93; *p* = 0.003 *; r = 0.33
Mean M	0.09 ± 0.61	0 ± 0	Z = −0.90; *p* = 0.367
Mean F	3.16 ± 3.72	2.46 ± 4.05	Z = −1.50; *p* = 0.134
Mean DMFT	7.02 ± 6.3	3.43 ± 4.12	Z = −2.92; *p* = 0.003 *; r = 0.33

* statistically significant.

**Table 3:** Differences in pufa/PUFA index and their components in the study and control groups.

	Control Group (n = 46)	Study Group (n = 40)	Statistical Significance
Mean p	0.36 ± 0.74	0.18 ± 0.50	Z = −0.68; *p* = 0.499
Mean u	0 ± 0	0 ± 0	Z = 0; *p* = 1
Mean f	0.5 ± 1.16	0.18 ± 0.59	Z = −1.03; *p* = 0.305
Mean a	0.14 ± 0.36	0 ± 0	Z = −1.78; *p* = 0.072; r = 0.26
Mean pufa	1.0 ± 1.66	0.36 ± 0.73	Z = −1.05; *p* = 0.293
Mean P	0.53 ± 1.24	0.03 ± 0.17	Z = −2.60; *p* = 0.009 *; r = 0.29
Mean U	0 ± 0	0 ± 0	Z = 0; *p* = 1
Mean F	0 ± 0	0 ± 0	Z = 0; *p* = 1
Mean A	0.05 ± 0.3	0 ± 0	Z = −0.92; *p* = 0.367
Mean PUFA	0.42 ± 1.17	0.03 ± 0.17	Z = −1.53; *p* = 0.126

* statistical significance.

**Table 4:** Salivary interleukin 6 and interleukin 18 concentration in the study and control groups.

	Control Group (n = 46)	Study Group (n = 40)	
Mean IL6 concentration (pg/mL)	13.11 ± 11.21	8.76 ± 9.09	*Z* = −2.44; *p* = 0.015 *; *r* = 0.27
Mean IL18 concentration (pg/mL)	43,857.05 ± 39,951.85	41,554.17± 36,609.52	*Z* = −0.15; *p* = 0.883

* statistically significant.

**Table 5:** Spearman’s correlation rank coefficients.

		ICDAS-II 1–2 Scores	Decayed Primary Teeth	dmft	Decayed Permanent Teeth	DMFT	Pulp/Pericapical Tissues Diseases
IL6 pg/mL	*r_s_*	0.15	0.04	0.06	0.28	0.18	0.26
	*p*	0.384	0.835	0.716	0.014 *	0.106	0.017 *
IL18 pg/mL	*r_s_*	0.52	0.28	0.14	0.18	0.16	0.29
	*p*	0.001 *	0.093	0.420	0.116	0.163	0.007 *

* statistical significance.

## Data Availability

The data that support the findings of this study are not openly available due to sensitivity reasons and are available from the corresponding author upon reasonable request. Data are located in controlled access data storage at the Medical University of Warsaw.
